# The Effects of Yuan-Zhi Decoction and Its Active Ingredients in Both *In Vivo* and *In Vitro* Models of Chronic Cerebral Hypoperfusion by Regulating the Levels of A*β* and Autophagy

**DOI:** 10.1155/2020/6807879

**Published:** 2020-02-22

**Authors:** Yan Liu, Xiaobo Huang, Wenqiang Chen, Yujing Chen, Ningqun Wang, Xiling Wu

**Affiliations:** Department of Traditional Chinese Medicine, Xuanwu Hospital, Capital Medical University, Beijing 100053, China

## Abstract

Chronic cerebral hypoperfusion (CCH) is closely related to the occurrence of Alzheimer's disease (AD) in the elderly. CCH can induce overactivation of autophagy, which increases the deposition of amyloid-*β* (A*β*) plaques in the brain, eventually impairing the cognitive function. Yuan-Zhi decoction (YZD) is a traditional Chinese medicine (TCM) formulation that is used to treat cognitive dysfunction in the elderly, but the specific mechanism is still unclear. In this study, we simulated CCH in a rat model through bilateral common carotid artery occlusion (BCCAO) and treated the animals with YZD. Standard neurological tests indicated that YZD significantly restored the impaired cognitive function after BCCAO in a dose-dependent manner. Furthermore, YZD also decreased the levels of A*β* aggregates and the autophagy-related proteins ATG5 and ATG12 in their hippocampus. An *in vitro* model of CCH was also established by exposing primary rat hippocampal neurons to hypoxia and hypoglycemia (H-H). YZD and its active ingredients increased the survival of these neurons and decreased the levels of A*β*1-40 and A*β*1-42, autophagy-related proteins Beclin-1 and LC3-II, and the APP secretases BACE1 and PS-1. Finally, both A*β* aggregates showed a positive statistical correlation with the expression levels of the above proteins. Taken together, YZD targets A*β*, autophagy, and APP-related secretases to protect the neurons from hypoxic-ischemic injury and restore cognitive function.

## 1. Introduction

Imaging studies show that Alzheimer's disease (AD) and vascular dementia (VD) patients exhibit hypoperfusion of the brain tissue [1]. Chronic cerebral hypoperfusion (CCH) is common among the elderly and occurs when the blood flow to the brain tissue is reduced, resulting in insufficient oxygen and glucose supply. This in turn leads to oxidative stress, apoptosis, autophagy, etc., culminating in nerve cell death in areas involved in cognitive function, such as the hippocampus [2]. Therefore, hypoperfusion of the brain tissue and the downstream damage are important pathological basis of both AD and VD. AD is a progressive neurodegenerative disease [3] characterized by neurotoxic amyloid-*β* (A*β*) plaque deposition, neurofibrillary tangles caused by tau hyperphosphorylation, and loss of neurons and synapses [4, 5]. However, the exact pathogenesis of AD is not fully understood.

Autophagy is a conserved pathway in eukaryotic cells, which recycles damaged organelles and macromolecules in order to maintain cellular homeostasis and metabolism [6]. Recent studies show that autophagy is closely related to the accumulation of A*β* aggregates and plays a pathological role in AD [7]. The affected neurons in AD show accumulation of autophagic vesicles (AVs) [8], which was initially attributed to increased autophagy but more recent evidence points to impaired autophagosome clearance [9]. Nevertheless, the interaction between A*β* and autophagy is highly complex. On the one hand, A*β* can be degraded by the autophagic machinery, and upregulation of autophagy has in fact been associated with reduced A*β* [10, 11]. On the other hand, A*β* can also be produced in autophagosomes [12].

The insufficient blood flow and the metabolic disorders in neurons and vascular endothelial cells following CCH lead to overactivation of autophagy [13]. This generates numerous AVs, wherein *β*-secretase cleaves the amyloid precursor protein (APP) to A*β*. In addition, a considerable number of AVs are not degraded by lysosomal fusion, resulting in their accumulation and impaired A*β* clearance [14]. Furthermore, ischemic injury increases the activity of *γ*-secretase in the neurons, which further increases the production of A*β* [15]. The latter accumulates in different brain regions, such as the hippocampus, resulting in massive loss of hippocampal neurons and reduced choline acetyltransferase activity. This in turn leads to the characteristic pathological, biochemical, and clinical manifestations of AD.

Despite improvements in our understanding of the pathological basis of AD, the current preventive and therapeutic measures are not effective [16]. Traditional Chinese medicine (TCM) has a long history of treating dementia and cognitive dysfunction. Numerous studies have shown that certain herbs and compounds in TCM formulations can improve the cognitive function of patients with mild to moderate AD [17–19]. Sporadic AD is more common in the elderly, and the TCM theory holds that the elderly are more debilitated and have insufficient kidney essence. This would result in blocked qi, blood, and body fluids, and the generation and aggregation of phlegm, conditions that are conducive to cognitive and memory decline. Yuan-Zhi decoction (YZD) is listed in the *General Medical Collection of Royal Benevolence* dating back to the Northern Song Dynasty for the treatment of cognitive dysfunction. It is a mixture of *Polygalae Radix* and *Acori Tatarinowii Rhizoma* that nourish the kidney essence and resolve the phlegm. A previous study [20] showed that YZD improved the learning and memory abilities of rats with diabetes-induced cognitive impairment by protecting the hippocampal neurons. In addition, a recent clinical study [21] with YZD as the main components showed that YZD effectively improved the cognitive function of patients with vascular mild cognitive impairment and had minimal adverse effects. It increased the serum levels of the antioxidant superoxide dismutase and reduced that of acetylcholinesterase, malondialdehyde, and A*β*1-42. However, the underlying mechanisms by which YZD improves cognitive dysfunction caused by cerebral hypoperfusion are unclear.

In this study, we analyzed the effects of YZD and its active ingredients in both *in vivo* and *in vitro* models of CCH in terms of behavior/cognition, A*β* accumulation, neuronal injury, and autophagy.

## 2. Materials and Methods

### 2.1. Animals

Fifty-two 12-week-old male Sprague-Dawley (SD) rats (weighing 250–280 g) were obtained from the Beijing Vital River Laboratory Animal Technology Co. Ltd. (Certificate Number: SCXK (Jing) 2016-0006, Beijing, China). The animals were housed in a controlled environment (12/12 h light/dark cycle, 60% ± 5% humidity, 20–25°C) and had free access to water and food. All animal protocols were approved by the Ethics Committee of Xuanwu Hospital of Capital Medical University, in line with the National Institute of Health Guide for the Care and Use of Laboratory Animals. All efforts were made to alleviate the suffering of animals and minimize their number.

### 2.2. Preparation of Yuan-Zhi Decoction (YZD)

*P. Radix* and *A. Tatarinowii Rhizoma* were purchased from and decocted at the Pharmacy Department of Xuanwu Hospital, Beijing, China. Based on a previous study [20] and the Chinese Pharmacopoeia [22], the ratio of both herbs was 1 : 1. Briefly, the herbs were immersed in 10-fold volumes of water for 30 min and decocted twice at 100°C for 30 min. The decoctions were mixed and concentrated to 0.5, 1, and 2 g/mL of herbs in a water bath. The drug solutions were stored at 4°C until use. Based on the conversion coefficient of 6.25 for adult and rats [23], the low, medium, and high doses of YZD were, respectively, calculated as 5 g/(kg • d), 10 g/(kg·d), and 20 g/(kg·d).

### 2.3. YZD Serum Preparation

Ten SD rats were intragastrically administered with high-dose YZD daily for 5 days. The animals were fasted after day 5 and given the last dose on the morning of day 6. Blood was collected from the abdominal aorta 2 h later and centrifuged at 3000 rpm for 20 min to separate the serum. The latter was then incubated in a 56°C water bath for 30 min and then stored at −20°C until use.

### 2.4. Chemicals and Reagents

Donepezil hydrochloride tablets were obtained from Eisai China Inc. (Shanghai, China), tenuigenin (TEN, purity >98.0%) from Meilunbio (Dalian, China), *β*-asarone (purity >98.0%) from Biopurify (Chengdu, China), and rapamycin and 3-methyladenine (3-MA) from Selleck (USA). The cell counting kit-8 (CCK-8) was obtained from Solarbio (Beijing, China) and the ELISA kit from R&D system (Shanghai, China).

### 2.5. Induction of CCH and Treatment Regimen

The bilateral common carotid artery occlusion (BCCAO) model was established to simulate human CCH [24, 25], which shares several characteristics with AD and VD [26]. The rats were acclimatized for 1 week before the experiment and fasted for 12 h before surgery, during which period water was withheld in the last 4 h. The animals were then randomized into the following 7 groups (*n* = 6 each): (1) control—no surgery, (2) untreated model—BCCAO surgery, (3) sham-operated—only dissection, (4) YZDH—BCCAO + high-dose YZD, (5) YZDM—BCCAO + medium-dose YZD, (6) YZDL—BCCAO + low-dose YZD, and (7) donepezil hydrochloride (DNZ)—BCCAO + DNZ 0.52 mg/(kg·d). DNZ is an acetylcholinesterase inhibitor that improves cognitive function [27] and was used as the positive control. All but the control group rats were anesthetized with 0.4% pentobarbital sodium (0.5 ml/100 g, i.p.) and fixed on the operating table in a supine position. The skin over the neck was depilated and disinfected, and a midline incision was made. The common carotid arteries were exposed and separated from the attached muscles, adjacent blood vessels, and nerves with a glass dissection tool, followed by double ligation with 4-0 silk sutures. The blood vessels were then cut between the two ligation points. The vessels were neither ligated nor cut in the sham-operated rats. The animals were given free access to food and water after surgery. One week after the surgery, the animals were intragastrically administered with the appropriate YZD dose at 1 mL/100 g daily for 4 weeks. The control, model, and sham-operated rats were given the same dose of saline.

### 2.6. Morris Water Maze (MWM) Test

The MWM test was performed after the treatment regimen to assess the spatial learning and memory capabilities using a previously described method with some modifications [28, 29]. The water maze consisted of a circular pool (150 cm in diameter and 40 cm in height) affixed with a camera and computer analysis system to record the motion of the rats. The pool was filled to a depth of 30 cm with water at 25 ± 1°C, divided into 4 quadrants (1, 2, 3, and 4), and an escape platform (10 cm × 10 cm) was placed in the 2^nd^ quadrant. A white platform was then submerged to about 1 cm depth, and milk was poured over the water to make it opaque in order to prevent the rats from seeing the platform. The hidden platform task was performed four times a day for five consecutive days. Briefly, the animals were let into the pool facing the wall, and the entry point was changed every day but the position of the platform was fixed throughout the test period. The test was automatically terminated after 120 s. If the rats were unable to find the platform within 120 s, they were gently placed on the platform and allowed to stay for 15 s. After each test, the rats were lightly dried and returned to their cages, and the interval between tests was 15 min. During each trial, the time required to reach the platform was measured as the escape latency, and the distance travelled by the rats to search was recorded. On the 6^th^ day, a probe trial was conducted by removing the platform. The rats were allowed to swim freely for 90 s in the pool, and the time spent in the original platform quadrant was recorded. Image analysis was performed using the HVS Image water maze software (HVS Image Ltd., UK).

### 2.7. Passive Avoidance Test

The learning and memory capabilities of the animals were evaluated by the passive avoidance test using a shuttle-box apparatus that consisted of an illuminated chamber and a dark chamber of the same size separated by a sliding door [30, 31]. The adaptation, training, and retention trials were conducted for three consecutive days on the 49^th^, 50^th^, and 51^st^ days. On the first day, the rats were placed in the apparatus to let them adapt. For training on the second day, each rat was placed individually in the illuminated chamber, and once it entered the dark chamber on account of its nocturnal nature, the sliding door was closed. A mild electric shock (40 V, 0.3 A, 2 s) was delivered to its feet through the floor grid, and the rat was returned to the cage immediately. In case an animal did not enter the dark chamber on its own, it was coaxed into the same. On the third day during the retention trial, each rat was placed in the illuminated chamber, and the time taken for the rat to step from the illuminated to the dark chamber (step-through latency) and the number of times it entered the dark chamber were recorded. If it did not enter the dark chamber within 300 s, the test was terminated and the step-through latency was recorded as 300 s.

### 2.8. Immunohistochemistry (IHC)

After the behavioral tests, the rats were anesthetized with 0.4% pentobarbital sodium (1 mL/100 g, i.p.), and 0.9% warm saline was perfused into the left ventricle, followed by 4% paraformaldehyde (pH 7.4) at 4°C. The brain tissues were removed immediately and immersed in 4% paraformaldehyde for 24 h. The fixed brains were embedded in paraffin and cut into 5 *μ*m-thick sections. For IHC, the sections were first deparaffinized with xylene, heated in citrate buffer (pH 6) for antigen retrieval, washed thrice with 10 mM PBS, and incubated with 3% H_2_O_2_ for 10 min at room temperature to quench the endogenous peroxidase. Following another PBS wash, the sections were blocked with 5% bovine serum albumin (BSA, Sigma, USA) for 30 min at room temperature, followed by overnight incubation with primary antibodies against A*β*1-40 (1 : 50; Merck, Germany), A*β*1-42 (1 : 50; Abcam, UK), ATG5 (1 : 500; Immunoway, USA), and ATG12 (1 : 500; Abcam, UK) at 4°C. The sections were washed and incubated with goat anti-rabbit IgG (ZSGB-BIO, Beijing, China) for 30 min at 37°C. The color was developed using a 3,3′-diaminobenzidine tetrahydrochloride (DAB, Beyotime Biotechnology, Beijing, China) kit, following which the sections were dehydrated in xylene and sealed with a neutral gum. For the negative controls, PBS was used instead of the primary antibody. The stained sections were observed under an optical microscope (ECLIPSE E600; Nikon, Japan) and the integrated optical density (IOD, % of control) of the stained areas was analyzed using the ImageJ software.

### 2.9. Hippocampal Neurons Isolation and Culture

Neonatal SD rats obtained from the Animal Center of the Chinese Academy of Medical Sciences were swabbed with 75% alcohol and sacrificed. The hippocampus was dissected, minced, and digested with 0.125% trypsin (Gibco, USA) at 37°C for 20 min. The tissue digest was then washed with DMEM/F12 (Gibco, USA) containing 10% FBS to stop trypsin activity, and the single cells were resuspended in the complete medium at the density of 1 × 10^6^/ml. The neurons were seeded in a 96-well plate coated with 0.1 mg/mL poly-l-lysine (Sigma, USA) and cultured at 37°C under 5% CO_2_ for 36 h. DMEM was then replaced with the Neurobasal medium (supplemented with 3 *μ*g/ml cytarabine (Sigma, USA) and 1 × B-27 (Gibco, USA)) to inhibit the meiotic growth of glial cells. The medium was completely replaced with fresh medium after 12 h, and half of the medium was replaced every 3 days for 14 days.

### 2.10. Hypoxia-Hypoglycemia Exposure and Treatment Regimen

**Figure 1:**
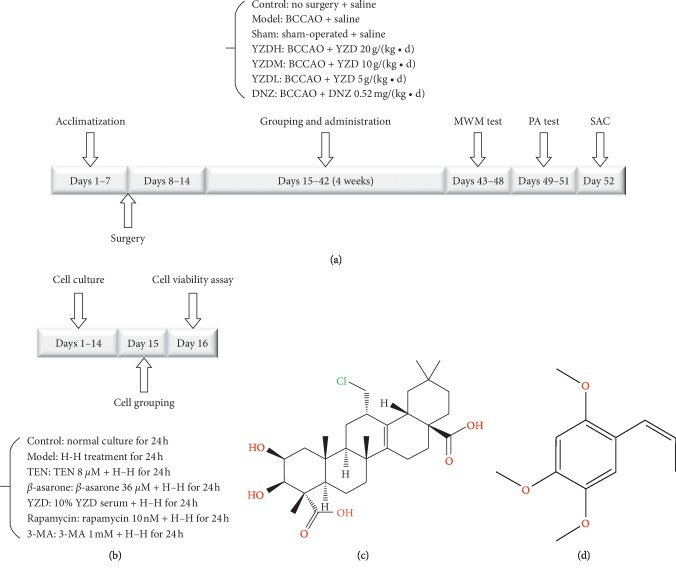
Experimental design of the *in vivo* (a) and *in vitro* (b) CCH models. Chemical structure of tenuigenin (c) and *β*-asarone (d). MWM, Morris water maze; PA, passive avoidance; SAC, sacrificed; control: control group; model: model group; sham: sham-operated group; YZDH: high-dose Yuan-Zhi decoction (20 g/(kg·d)); YZDM: medium-dose Yuan-Zhi decoction (10 g/(kg·d)); YZDL: low-dose Yuan-Zhi decoction (5 g/(kg·d)); DNZ: donepezil hydrochloride; H-H, hypoxia-hypoglycemia; TEN: tenuigenin; 3-MA: 3-methyladenine.

Hippocampal neurons were exposed to hypoxic-hypoglycemic (H-H) conditions for 24 h to simulate CCH *in vitro* [32]. The cells were cultured with hypoglycemic DMEM (containing 1000 mg/L glucose, Gibco, USA) at 37°C in a closed container, wherein a mixture of 2.52% O_2_, 5.02% CO_2_, and 92.46% N_2_ was continuously flushed to create a hypoxic environment. The H-H conditions were induced for 24 h, during which period the neurons were treated with the following reagents: (1) untreated (model), (2) 8 *μ*M tenuigenin (TEN), (3) 36 *μ*M *β*-asarone, (4) 10% YZD serum, (5) 10 nM rapamycin, and (6) 1 mM 3-methyladenine (3-MA). TEN and *β*-asarone are the respective main active ingredients of *P. Radix* and *A. Tatarinowii Rhizoma* (Figure 1). Rapamycin is a promoter of autophagy [33], while 3-MA is an inhibitor [34]. The doses of TEN, *β*-asarone, YZD serum, rapamycin, and 3-MA were determined according to our previous experiments. An untreated control cultured in normal medium was also included. Eight replicates were tested for each group, and the experiment was repeated thrice.

### 2.11. Cell Viability Assay

Cell viability was measured using the cell counting kit-8 (CCK-8) according to the manufacturer's instructions. Following the appropriate treatments, 10 *μ*L CCK-8 solution was added to each well, and the cells were incubated at 37°C for 4 h. The absorbance of each well at 450 nm was measured using a microplate reader. Each well was measured thrice, and the average was calculated. The survival rate of hippocampal neurons (%) was calculated relative to that of the control.

### 2.12. ELISA

The levels of A*β*1-40 and A*β*1-42 in the lysates of hippocampal neurons were tested using a sandwich ELISA kit according to the instructions.

### 2.13. Immunofluorescence

The cells were cultured in a 96-well plate, fixed using a fixation buffer (BD, USA) for 15 min, and washed thrice with PBS. After permeabilization with 0.5% Triton X-100 (diluted with PBS) for 20 min at room temperature, the cells were washed and blocked with 5% BSA for 30 min. The cells were then incubated overnight with anti-Beclin-1 (1 : 200; ImmunoWay, USA) and anti-LC3-II (1 : 200; ImmunoWay, USA) antibodies at 4°C. After washing with PBS, the secondary antibodies (goat anti-mouse IgG H&L or goat anti-rabbit IgG H&L (Alexa Fluor® 488) 1 : 500; Abcam, UK) were added, and the cells were incubated for 60 min in the dark. The stained cells were washed 5 times with PBS, counterstained with 4′,6‐diamidino‐2‐phenylindole (DAPI, 1 : 200; Gibco, USA) for 15 min in the dark, and washed again. The cells were observed under an inverted fluorescence microscope (TE2000; Nikon, Japan), and the fluorescence intensities (% of control) were analyzed using ImageJ software.

### 2.14. Western Blotting

The cells were lysed in the suitable buffer, and the lysates were centrifuged at 13,000 rpm for 20 min at 4°C. The protein concentration was measured using a BCA kit (Beyotime Biotechnology, Shanghai, China). Equal amounts of protein (10 *μ*g) from each sample were boiled in the loading buffer for 5 min and resolved by 12% SDS-PAGE. The protein bands were transferred onto an NC membrane (0.45 *μ*m pore size; Millipore, USA) that was blocked in 3% BSA-TBST for 30 min, and then incubated overnight with mouse anti-Beclin-1 (1 : 2000; Immunoway, USA), rabbit anti-LC3-II (1 : 2000; Immunoway, USA), mouse anti-BACE1 (1 : 1000; Immunoway, USA), rabbit anti-PS-1 (1 : 2000; Immunoway, USA), and rabbit anti-ADAM17 (1 : 1000; Abcam, UK) primary antibodies at 4°C. After washing 5 times with TBST, the membrane was incubated with HRP-conjugated goat anti-rabbit IgG (*H* + *L*) or goat anti-mouse IgG (*H* + *L*) (1 : 10000; Beyotime, Shanghai, China) secondary antibodies for 40 min at room temperature. The blots were washed again, developed using an ECL reagent (Millipore, USA) for 5 min, and the film was exposed and photographed. GAPDH was used as the internal control. The bands were scanned, and the relative gray values were calculated using ImageJ software.

### 2.15. Statistical Analysis

Statistical analysis was performed using SPSS Statistics 23.0 (IBM, USA). Data were presented as mean ± standard deviation (SD). Multiple groups were compared by the one-way analysis of variance (ANOVA) followed by the least significant difference (LSD) test. The correlation of A*β* with the autophagy markers and APP secretases were analyzed by linear correlation analysis using the correlation coefficient *R*^2^. *P* values <0.05 were considered statistically significant.

## 3. Results

### 3.1. YZD Restores the Cognitive Function of Rats after BCCAO

**Figure 2:**
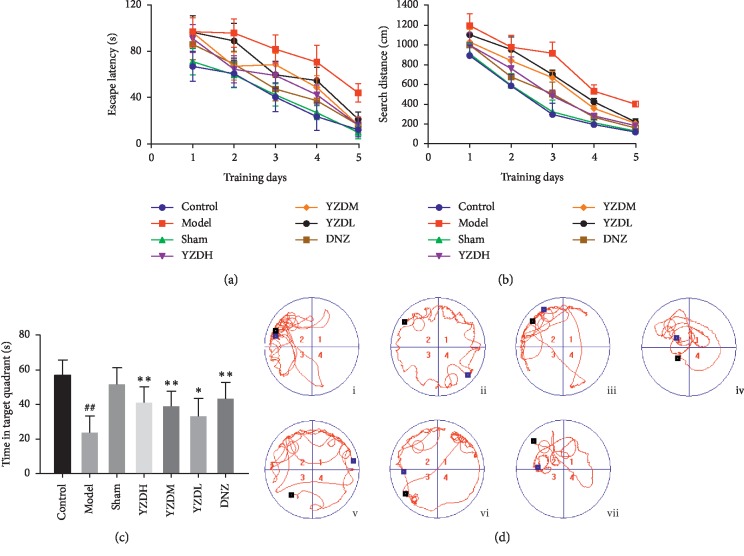
Morris water maze test evaluating spatial learning and memory ability. Escape latency (a) and search distance (b) in the hidden platform task. (c) Time spent in the target quadrant in the probe trial. (d) Representative swimming paths of spatial probe trial. 1, 2, 3, and 4 represent the 4 quadrants, respectively, the blue circle is the location of the original platform, the black square is the entry point for the rat, and the blue square is the exit point. Data are presented as mean ± SD (*n* = 5–6). ^##^*P* < 0.01, vs control group; ^*∗*^*P* < 0.05, ^*∗∗*^*P* < 0.01, vs model group. i/control: control group, ii/model: model group, iii/sham: sham-operated group, iv/YZDH: high-dose Yuan-Zhi decoction group (20 g/(kg·d)), v/YZDM: medium-dose Yuan-Zhi decoction group (10 g/(kg·d)), vi/YZDL: low-dose Yuan-Zhi decoction group (5 g/(kg·d)), vii/DNZ: donepezil hydrochloride group.

**Figure 3:**
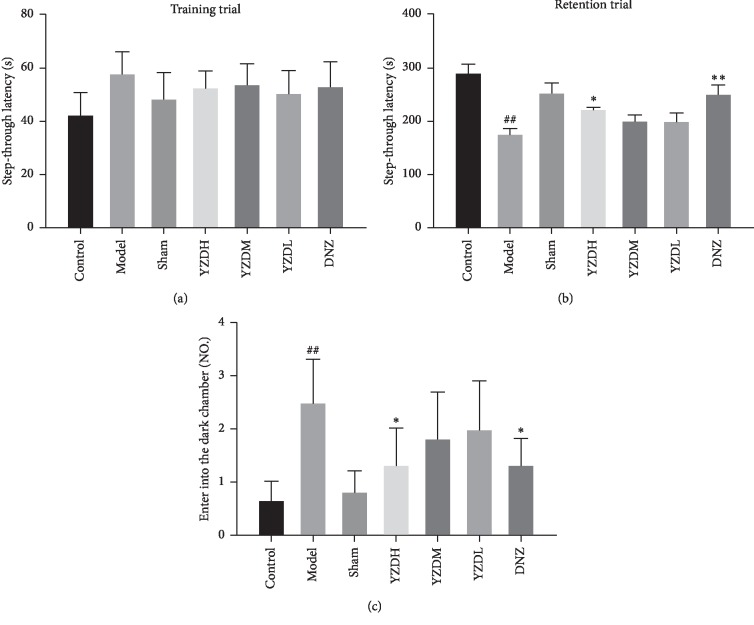
Passive avoidance test learning and memory ability of rats. Step-through latency (a, b) and number of times the rats entered into the dark chamber (c). Data are presented as mean ± SD (*n* = 5–6). ^##^*P* < 0.01, vs control group; ^*∗*^*P* < 0.05, ^*∗∗*^*P* < 0.01, vs model group.

Of the 36 rats that underwent BCCAO surgery, 2 (6%) died during the experiment. Some of the surviving rats showed transient reduction in appetite and modest weight loss, and none showed hemiplegia symptoms. BCCAO significantly increased the escape latency and search distance in the hidden platform task compared with the control and sham-operated groups (*P* < 0.01). On the second day, YZDH, YZDM, and donepezil significantly shortened the escape latency (*P* < 0.01) and search distance (*P* < 0.05) compared with the model group. From the third day to fifth day, YZDH, YZDM, YZDL, and donepezil significantly shortened both escape latency and search distance (*P* < 0.05 or *P* < 0.01; Figures 2(a) and 2(b)). There were no significant differences between the intervention groups. Furthermore, the time spent in the target quadrant in the probe trial was significantly shorter in the untreated model group compared to the control and sham-operated groups (*P* < 0.01) and restored by YZD and donepezil (*P* < 0.05 or *P* < 0.01; Figures 2(c) and 2(d)). In the passive avoidance test, the groups showed no difference during the training period (*P*=0.784), but the step-through latency in the retention period was significantly shorter and the number of ingresses into the dark chamber was greater in the untreated model group compared to the control and sham-operated groups (*P* < 0.01). YZDH and donepezil significantly prolonged the step-through latency of the rats (*P* < 0.05 or *P* < 0.01) and reduced the number of times they entered into the dark chamber (*P* < 0.05; Figure 3). Taken together, YZD significantly improved the spatial learning and memory ability of the rats after BCCAO.

### 3.2. The Effects of YZD on the Expression of Amyloid Aggregates and Autophagy-Related Proteins in Hippocampus after BCCAO

**Figure 4:**
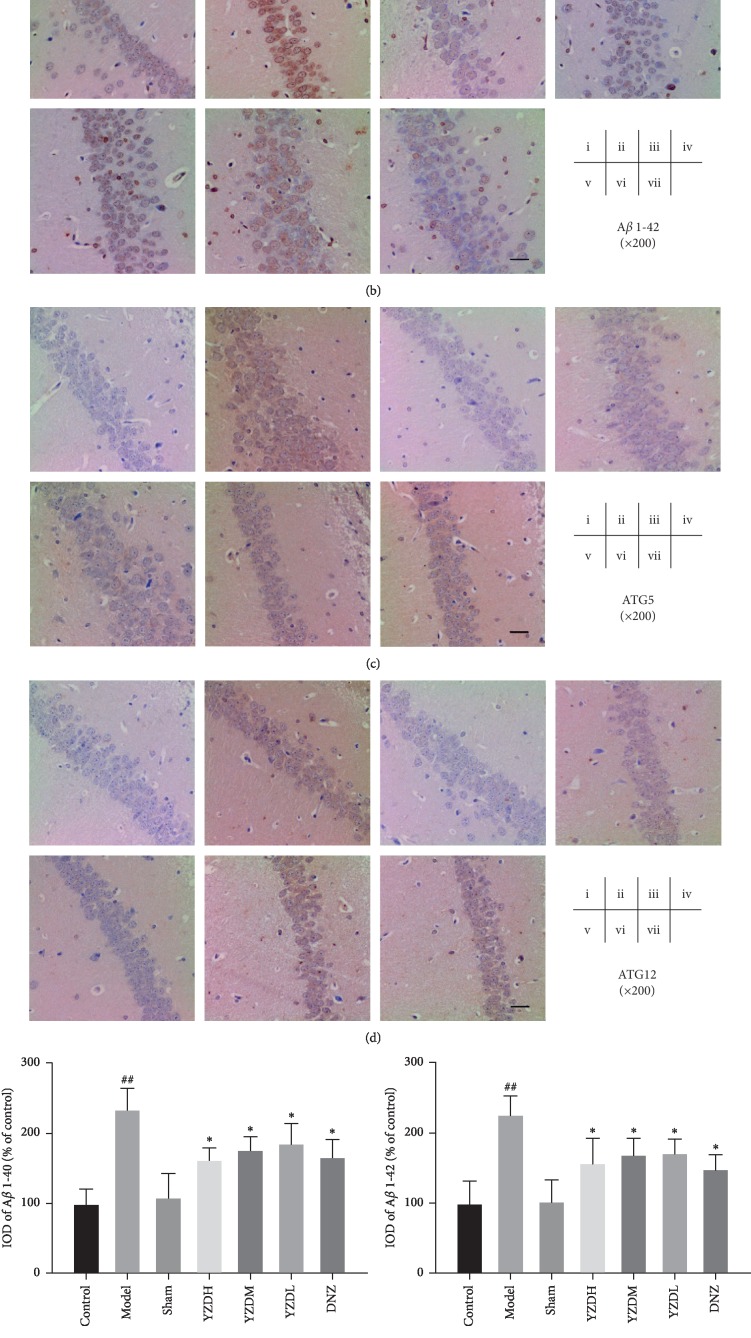
Effect of YZD on A*β* aggregate deposition and autophagy-related protein levels in the hippocampus. (a–d) Representative IHC images of the hippocampal CA1 region showing *in situ* expression of A*β*1-40, A*β*1-42, ATG5, and ATG12 (magnification: ×200). Scale bar = 50 *μ*m. (e–h) Quantification of A*β*1-40, A*β*1-42, ATG5, and ATG12 in the hippocampus (% of Control). Data are presented as mean ± SD (*n* = 5–6). ^#^*P* < 0.05, ^##^*P* < 0.01, vs control group; ^*∗*^*P* < 0.05, vs model group.

To determine the possible underlying mechanisms of the protective effects of YZD after BCCAO, we next analyzed the *in situ* levels of amyloid aggregates and autophagy-related proteins in the hippocampus. BCCAO significantly increased the accumulation of A*β*1-40 and A*β*1-42 in the hippocampal tissues compared to the control and sham-operated groups (*P* < 0.01), which was decreased by YZD and donepezil (*P* < 0.05; Figures 4(a), 4(b), 4(e), and 4(f)). The autophagy-related proteins ATG5 and ATG12 [35] were also significantly increased in the hippocampus of the untreated model group compared to the control and sham-operated groups (*P* < 0.05) and restored to baseline levels by YZD (*P* < 0.05) but not by donepezil intervention (Figures 4(c), 4(d), 4(g), and 4(h)).

### 3.3. YZD and Its Active Ingredients Protect Primary Hippocampal Neurons against H-H Injury

**Figure 5:**
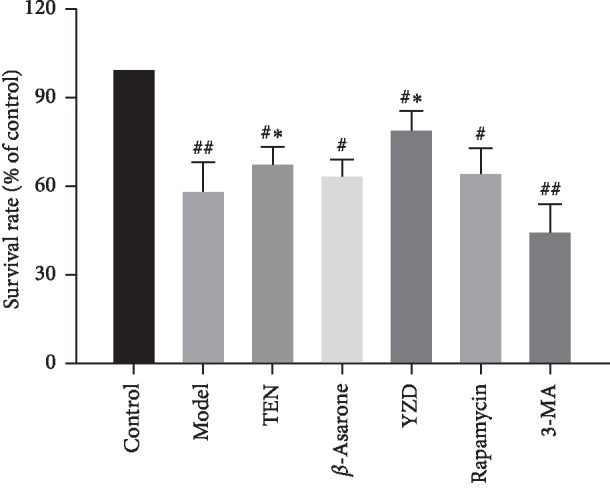
The survival rate of hippocampal neurons after hypoxia-hypoglycemia (% of Control) and different drug interventions. Data are presented as mean ± SD. ^#^*P* < 0.05, ^##^*P* < 0.01, vs control group; ^*∗*^*P* < 0.05, vs model group.

**Figure 6:**
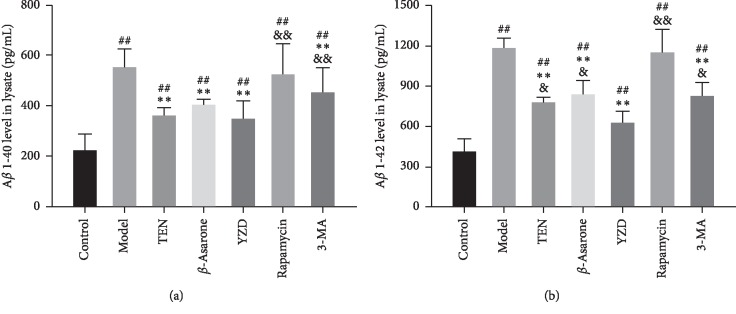
A*β*1-40 and A*β*1-42 levels in the lysates of hippocampal neurons. Data are presented as mean ± SD. ^##^*P* < 0.01, vs control group; ^*∗∗*^*P* < 0.01, vs model group; ^&^*P* < 0.05, ^&&^*P* < 0.01, vs YZD group.

**Figure 7:**
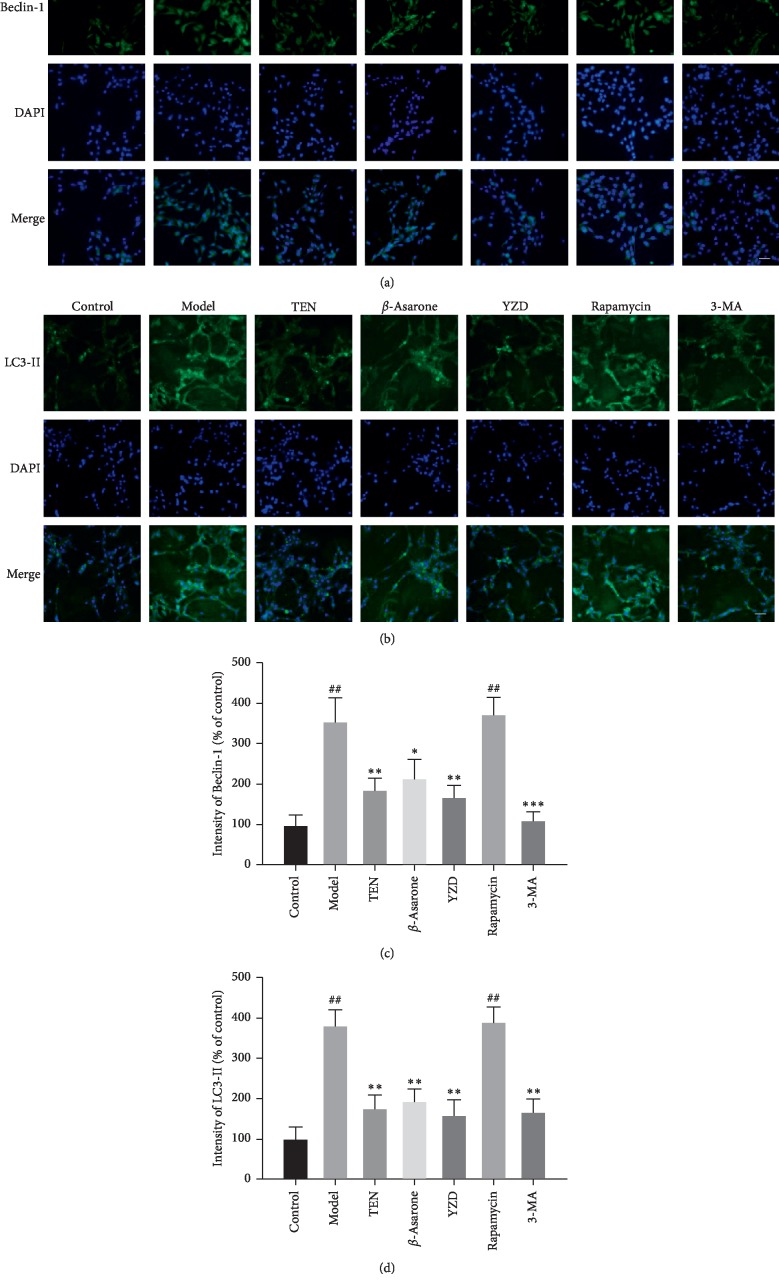
The expression of Beclin-1 and LC3-II in the hippocampal neurons. Representative immunofluorescence images showing *in situ* expression of Beclin-1 (a) and LC3-II (b) in the differentially treated hippocampal neurons (magnification: ×100). Scale bar = 100 *μ*m. Quantification of Beclin-1 (c) and LC3-II (d) in the hippocampal neurons in each group (% of Control). Data are presented as mean ± SD. ^##^*P* < 0.01, vs control group; ^*∗*^*P* < 0.05, ^*∗∗*^*P* < 0.01, ^*∗∗∗*^*P* < 0.001, vs model group.

**Figure 8:**
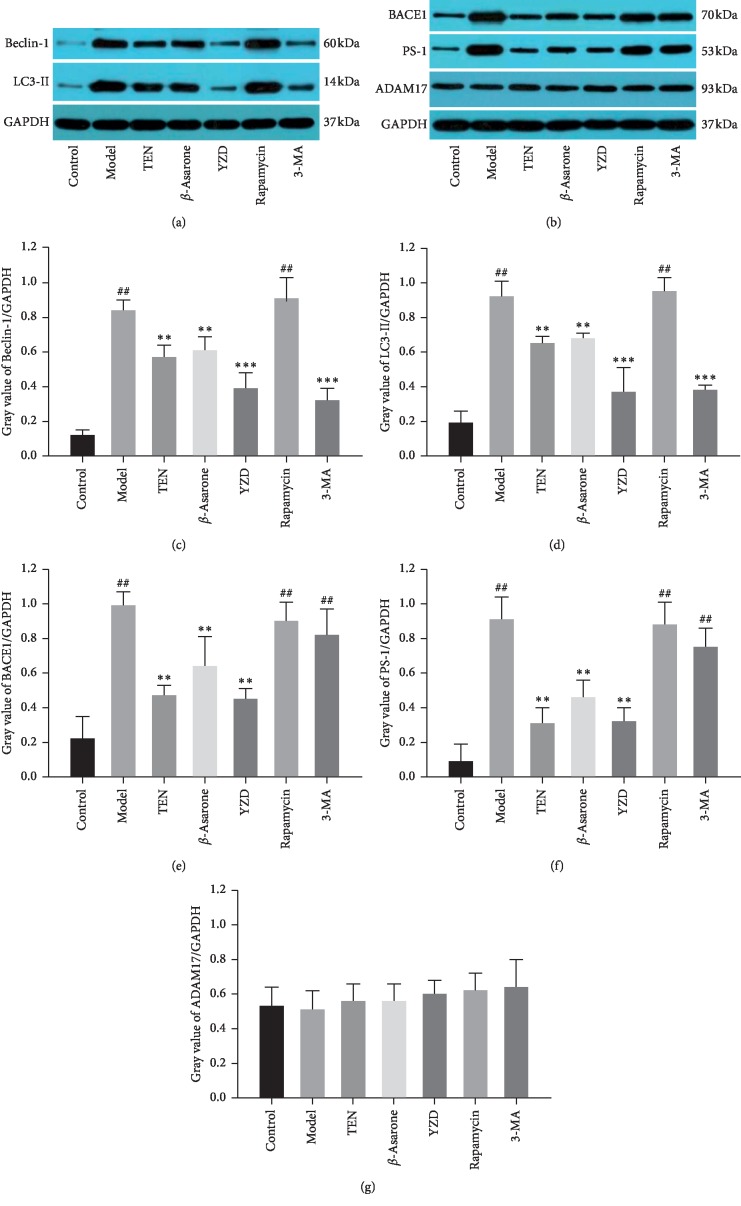
The expression levels of Beclin-1, LC3-II, BACE1, PS-1, and ADAM17 in hippocampal neurons. Data are presented as mean ± SD. ^##^*P* < 0.01, vs control group; ^*∗∗*^*P* < 0.01, ^*∗∗∗*^*P* < 0.001, vs model group.

The survival rate of hippocampal neurons exposed to hypoxia and hypoglycemia (H–H) decreased significantly compared to the control (*P* < 0.01 or *P* < 0.05) and was restored by TEN and YZD serum (*P* < 0.05). However, *β*-asarone intervention had no significant effect on the viability of these neurons (Figure 5). Furthermore, the levels of A*β*1-40 and A*β*1-42 in the lysates of injured hippocampal neurons were significantly higher compared to the control (*P* < 0.01) and decreased by TEN, *β*-asarone, YZD serum, and 3-MA (*P* < 0.01 for all; compared to the model group). Interestingly, the YZD serum had a greater ameliorative effect on A*β*1-40 compared to rapamycin and 3-MA (*P* < 0.01), as well as on A*β*1-42 compared to TEN, *β*-asarone, rapamycin, and 3-MA (*P* < 0.05 or *P* < 0.01; Figure 6). The expression levels of the autophagy flux indicators Beclin-1 and LC3-II [36–38] also increased following H-H exposure and rapamycin intervention compared to the control (*P* < 0.01) and were decreased by TEN, *β*-asarone, YZD serum, and 3-MA (*P* < 0.05 or *P* < 0.01 or *P* < 0.001; compared to the model group; Figure 7). Similar results were obtained with Western blotting (Figures 8(a), 8(c), and 8(d)). The APP secretases-related BACE1, PS-1, and ADAM17 [15, 39] were also analyzed, and while BACE1 and PS-1 were upregulated in the model, rapamycin, and 3-MA intervention groups (*P* < 0.01) and decreased by TEN, *β*-asarone, and YZD serum (*P* < 0.01), ADAM17 levels were similar among the different groups (Figures 8(b), 8(e), 8(f), and 8(g)).

### 3.4. The A*β* Protein Aggregates Are Correlated to the Autophagy and APP Secretases-Related Proteins

**Figure 9:**
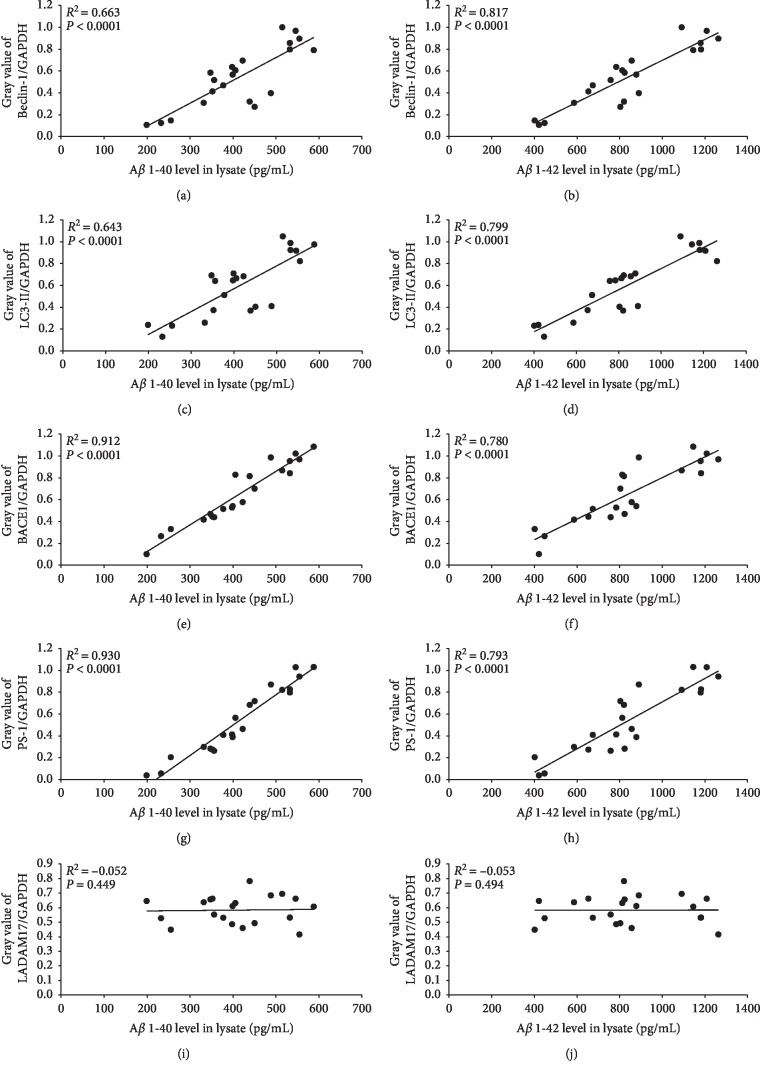
Correlation analysis between A*β*1-40/A*β*1-42 and the autophagy-related (Beclin-1 and LC3-II) and APP secretases-related proteins (BACE1, PS-1, and ADAM17) levels in hippocampal neurons. *R*^2^ is the correlation coefficient.

The expression levels of A*β*1-40 and Beclin-1 in the lysates of hippocampal neurons showed a Pearson correlation coefficient (*R*^2^) of 0.663 (*P* < 0.0001; Figure 9(a)) and that of A*β*1-42 and Beclin-1 showed an *R*^2^ of 0.817 (*P* < 0.0001; Figure 9(b)). Similar positive correlation was seen between A*β*1-40 and LC3-II (*R*^2^ = 0.643; *P* < 0.0001; Figure 9(c)), and between A*β*1-42 and LC3-II (*R*^2^ = 0.799, *P* < 0.0001; Figure 9(d)). Furthermore, BACE1 also showed a positive correlation with A*β*1-40 (*R*^2^ = 0.912; *P* < 0.0001; Figure 9(e)) and A*β*1-42 (*R*^2^ = 0.780, *P* < 0.0001; Figure 9(f)), as did PS-1 with both aggregates (A*β*1-40: *R*^2^ = 0.930; *P* < 0.0001; Figure 9(g), and A*β*1-42: *R*^2^ = 0.793; *P* < 0.0001; Figure 9(h)). However, ADAM17 failed to show any correlation with either A*β*1-40 (*R*^2^ = −0.052; *P*=0.449; Figure 9(i)) or A*β*1-42 (*R*^2^ = −0.053; *P*=0.494; Figure 9(j)).

## 4. Discussion

CCH, a common condition in the elderly, is closely related to the development of cognitive dysfunction [40]. In this study, we simulated CCH in a rat model using the BCCAO approach and treated the animals with varying doses of YZD, which significantly improved the learning and memory abilities of the rats in a dose-dependent manner, and markedly decreased neuronal A*β* accumulation in the hippocampus. In the *in vitro* simulation as well, YZD increased the survival rate of primary hippocampal neurons exposed to hypoxic and hypoglycemic conditions and decreased the expression levels of A*β*1-40 and A*β*1-42. Recent studies have shown a critical pathological role of autophagy in AD initiation and progression. Autophagy is initiated with the formation of autophagosomes by ATG5 and ATG12 [35], followed by recruitment of other proteins to the phagosome membrane by Beclin-1 [36], and finally the elongation and maturation of autophagosomes into autophagic vacuoles (AVs) that is regulated by LC3-II [37, 38]. We found that all the above markers of autophagy flux were upregulated following the hypoxic-ischemic stimulus both *in vivo* and *in vitro* and were decreased by YZD and its active ingredients. Thus, we surmised that YZD can inhibit the overactivation of autophagy caused by hypoxia-ischemia.

A*β* is formed by the sequential cleavage of APP by *β*-secretase (BACE) and a *γ*-secretase complex consisting of presenilin (PS), nicastrin, APH-1, and PEN-2 [15]. APP can also be cleaved inside the A*β* domain by *α*-secretase, which is regulated by ADAM17. Enhanced ADAM17 activity increases secretion of the neuroprotective soluble APP *α* fragment and decreases A*β* production, which may be beneficial for AD [39]. A*β* is produced and stored in AVs [41, 42], where it can be generated and degraded to maintain homeostasis [43]. CCH impairs the function of the blood-brain barrier and decreases A*β* clearance [44], which leads to overactivation of autophagy and a massive increase in AVs that overwhelms the lysosomes, eventually resulting in A*β* accumulation in the AVs [43]. Furthermore, the hypoxia-ischemia exposure also promotes the hydrolysis of APP by activating *β*- and *γ*-secretase and stimulates the production of A*β* [45]. Deposition of the A*β* aggregates and neuronal death in brain regions associated with cognition, such as hippocampus, leads to memory loss and dementia [44]. Therefore, we also examined the expression of APP-related secretases in the injured hippocampal neurons. YZD and its active ingredients (TEN and *β*-asarone) significantly reduced the expression of BACE1 and PS-1 that was increased upon hypoxic-ischemic simulation, while that of ADAM17 was unaffected. In addition, the A*β* protein aggregates were positively correlated to the autophagy-related and APP secretases-related proteins except ADAM17. Therefore, hypoxia/ischemia-induced A*β* accumulation in the hippocampal neurons depends on the *β*- and *γ*-secretases, which could also be the potential targets of YZD.

YZD is a mixture of *P. Radix* and *A. Tatarinowii Rhizoma*, and the formulation and its components have a long history of treating dementia [46]. Tenuigenin (TEN) and *β*-asarone, the respective main active ingredients of *P. Radix* and *A. Tatarinowii Rhizoma*, can enhance the learning and memory of animals, protect hippocampal neurons, and inhibit autophagy [29, 47]. In this study, we observed a potent effect of YZD compared to the individual active ingredients, which is likely due to the synergistic effects of the active ingredients and the multiple pathways of action. Further studies are needed to determine whether YZD targets the upstream pathways of autophagy, and to analyze the mechanism through which YZD improves CCH-induced cognitive dysfunction.

## 5. Conclusion

YZD significantly improved the cognitive abilities of rats after BCCAO and increased the survival of hippocampal neurons by inhibiting autophagy and the APP-related *β*- and *γ*-secretases, which in turn prevented A*β* deposition.

## Data Availability

The data used to support the findings of this study are available from the corresponding author upon request.
